# Assessing the Mental Health of Brazilian Students Involved in Risky Behaviors

**DOI:** 10.3390/ijerph17103647

**Published:** 2020-05-22

**Authors:** Daise Fernanda Santos Souza Escobar, Priscilla Rayanne e Silva Noll, Thaís Ferreira de Jesus, Matias Noll

**Affiliations:** 1 Public Health, Instituto Federal Goiano—Campus Ceres, Ceres 76300-000, Brazil; daise.ifgoianoceres@gmail.com (D.F.S.S.E.); priscilla.silva@ifgoiano.edu.br (P.R.eS.N.); thaisferreiradejesus@hotmail.com (T.F.d.J.); 2 Department of Obstetrics and Gynecology, Faculdade de Medicina, Universidade de São Paulo, São Paulo 05508-070, Brazil

**Keywords:** adolescents, risky behaviors, school, substance abuse, mental health

## Abstract

Adolescence, which is the transition from childhood to adulthood, is marked by emotional sensitivity and inconsistency and may be affected by mental health problems. In order to fill the gap related to the risky behaviors in students in Brazil, our cross-sectional study aimed to analyze the relationship between risky behaviors and indicators of mental health of Brazilian students. We used the data from the National School Health Survey to analyze the relationship between risk behaviors and three symptoms of mental health issues: feeling of being alone, number of close friends, and trouble sleeping due to worries. The sample consisted of 102,072 students in Brazil (48.3% boys and 51.7% girls), aged between 11 to 19 years. The risk behaviors evaluated were substance use, sedentary lifestyle, sexual behavior, and suffering violence and bullying. We have performed a multivariate analysis based on the Poisson regression model, and the measure of effect used was the prevalence ratio (PR) with confidence intervals (CI) of 95%. Our results showed that students with symptoms of mental health issues were involved in risky behaviors, including drug use and unsafe sex. Thus, mental illness outcomes may be associated with risky behaviors, or mental health may be impaired by them. Given these findings, in-school programs focused on improving mental health outcomes should be developed.

## 1. Introduction

Mental health is a subject of extreme social importance [1,2,3,4]. For individuals with low socioeconomic status, poor mental health outcomes may also lead to further issues in their daily lives [1,4,5,6]. To address this, countries such as the United States, the United Kingdom, and France have invested resources into their mental health systems [7]. In Brazil, with the use of the World Health Organization (WHO) guidelines and the creation of The Mental Health Policy in 2001, increased mental health services have begun to allow discussions around the subject, as well as provide better treatment conditions [7]. However, although many improvements in the field of mental health have been achieved, the relevant amount of population studies conducted in Brazil are insufficient when compared to that of other developed countries, indicating a need for more research focused on mental health [8]. Furthermore, as of 2001, more than 90% of countries globally did not include adolescents in their mental health policies, even though this age group is often most affected by mental health disorders [1].

During adolescence, individuals experience developmental changes [9,10,11]. The transition from childhood to adulthood is marked by emotional sensitivity and inconsistency, which can lead to intensified feelings [2,9,12]. This can also contribute to mental health disorders and interfere with positive decision-making skills [10,13]. Hence, episodes of rebellion and dangerous habits, such as substance abuse and violent behavior, may start to develop [9,11,13].

A study [14] conducted with 2227 students in grades six through eight in North Carolina, United States, demonstrated that substance use was associated with attempting to be included and accepted by their peers and other members of social groups. Hence, the poor state of mental health was considered a factor that could lead to drug use. Other studies [10,13,15] conducted on adolescents have shown similar results that link substance use with poor mental health outcomes. Addiction has also been linked to poor mental health outcomes, including the feeling of being alone and trouble sleeping, especially when the substances, such as nicotine or alcohol, can create a chemical dependence [16,17].

Alcohol is the most accessible illicit substance in Brazil. It is common for Brazilian adolescents to have access to both alcoholic beverages and cigarettes, despite laws that prohibit the sale of these products to minors [16]. Although Brazil has public policies aimed at preventing smoking and alcohol use, alcohol and tobacco are still widely consumed by adults and adolescents, especially by those who experience social vulnerability and by children whose parents smoke [10]. The same applies to alcohol use; children of alcohol-dependent parents are more susceptible to becoming alcohol-dependent too [18]. Furthermore, alcohol is a substance considered socially acceptable in Brazil, even among minors. In addition, drug use and other risky behaviors can increase the risk of poor mental health outcomes [10]. There is also an association between alcohol consumption and smoking cigarettes, along with other drugs [10,19], unsafe sexual practices [20], and violent behaviors [10].

Being a victim of physical aggression or of bullying [21] is related to poor mental health results in many communities, including the ones in Brazil [14,20]. Unfortunately, it is also common for young people with mental health disorders to engage in premature sexual activity and participate in other harmful activities in an attempt to escape their problems [10]. In addition to the personal impact, social consequences are also associated with these risky behaviors; low professional productivity, behavioral problems, and the economic cost of healthcare paid by the country and families continue from adolescence to adulthood [22,23]. Other problems are school-related damages, such as poor grades, dropouts, bullying and its consequences [20,24], sedentarism, [25] and risky sexual behaviors [20]. We realize it is necessary to understand whether there is an association between mental health and the adoption of risky behaviors in students in Brazil.

Adolescents spend a lot of time in school and establish a significant amount of their interpersonal relationships in this environment. Therefore, schools are an important data source [21]. Thus, our study aimed to analyze the relationship between risky behaviors and three indicators of mental health of Brazilian students. Our specific objectives were to verify the association between the aforementioned three mental health indicators and (a) substance use, (b) sexual behavior, (c) sedentarism, (d) suffered bullying, and (e) suffered physical violence.

We have evaluated mental health through three indicators: (a) feeling alone, (b) lacking close friends, and (c) having trouble sleeping due to worries. As our study is a populational study, we used these three variables as indicators of mental health [20], not as psychiatric disorder exams. According to WHO [26], these indicators are fundamental for the promotion of mental health, which is related to the capacity for a healthy personal and social relationship. Therefore, we do not diagnose the mental health of adolescents; we only check for the presence of these symptoms, which are related to insomnia and loneliness and indicate poor mental health outcomes [1,27]. We have hypothesized that risky behaviors, such as frequent use of cigarettes, drugs and alcohol consumption, risky sexual behavior, sedentarism, and suffered bullying, are higher indicators of poor mental health.

## 2. Materials and Methods

### 2.1. Type of Study

We conducted a cross-sectional study using data from the National School Health Survey (PeNSE) database, a survey conducted in partnership between the Brazilian Institute of Geography and Statistics (https://www.ibge.gov.br/) and the ministries of health (http://www.saude.gov.br) and of education (http://portal.mec.gov.br/). In 2015, the PeNSE was approved by the National Health Council and by the National Commission of Ethics in Research (CONEP, protocol n. 1.006.467 of 30.03.2015), which regularizes and approves Brazilian research involving human beings [28].

### 2.2. Population

The PeNSE sample was dimensioned to infer population parameters of the geographic domains analyzed. These domains included the five major geographical regions in Brazil (North, Northeast, Midwest, Southeast, and South), as well as the 26 Federative Units of Brazil and the Federal District, including capitals and municipalities. The municipal samples and the geographical strata of the capitals were obtained randomly and with equal probability and were calculated with the following parameters: a prevalence ratio of 0.5, a maximum error of 0.03%, and a confidence interval of 95%. A class sample was obtained for each school and an independent sample of students for each stratum. The sample consisted of 120,122 students from 4159 classes of 3040 schools. Sample loss, owing to the total number of absent students, was 14.8%. The final sample included 102,072 students who were present on the day of data collection and participated in the study [28].

### 2.3. Data Collection and Analysis Procedures

The data collection for the version of the PeNSE used in this study occurred between April and September 2015. The PeNSE used self-report questionnaires delivered to students by using smartphones. The technicians distributed the smartphones to students present in the classroom on the day of data collection and explained how to use the device. All students in the sampled classes were invited to answer the questionnaires, and those who agreed to participate completed an informed consent form (ICF). PeNSE data collection assesses various health and quality of life outcomes, and several studies are dedicated to analyzing these aspects based on their results [29,30,31,32,33].

As it is a population-based survey, PeNSE evaluated broad issues related to insomnia and loneliness [27,34,35] through behaviors such as feeling alone, having no more than one close friend, and difficulty sleeping [12,27]. Then, indicators of mental health were assessed using the following objective-type questions (Table 1):

These questions were asked because, although such events may occur many times during a lifetime, when combined and recurrent, they are signs of mental illness [36]. We also evaluated sociodemographic characteristics (gender, age, and mother’s education).

According to the definition of risk behaviors [37], the following risky behavioral factors were considered to be exposure variables (Table 2):

The data was analyzed using descriptive statistics and the Wald chi-square association test (bivariate analysis) for the three variables regarding mental health. The following risk behavioral factors were considered independent variables: use of cigarettes and illicit drugs, alcohol consumption, age at onset of sexual activity, condom use, incidence of being bullied and/or experiencing aggression, and sedentarism. Independent variables with a significance level of *p* < 0.2 in the bivariate analysis were included in an adjusted analysis with sociodemographic variables (gender, age, and mother’s level of education) through the Poisson regression model with robust variance. The measure effect was the prevalence ratio (PR) (α = 0.05). The statistical analyses were performed using the Statistical Package for the Social Sciences (SPSS 23) (IBM, Armonk, NY, USA).

## 3. Results

In total, 102,072 students answered the questionnaire—of which, 49,290 were male and 52,782 were female. Our results indicated that female adolescents feel more alone (55.9%), are more likely to have no more than one friend (10.7%), and have more difficulties falling asleep due to worries (45.1%) than male adolescents (32.7%, 10.3%, and 26.7%, respectively) (Table 3). With respect to age, our results revealed that the older students were more likely to feel alone (47.0%), have trouble sleeping due to worries (41.6%), and have fewer friends (13.9%) (Table 3). The mother’s level of education influenced all the variables, indicating that the lower the education level of their maternal figures, the more likely students are to feel alone (47.6%), have fewer friends (14.6%), and have trouble sleeping due to more frequent worries (41.8%) (Table 3).

The results of the bivariate analysis indicated that mental health indicators are associated with smoking cigarettes, drinking alcoholic beverages, drug use (Table 4), early sexual intercourse, no use of condoms (Table 5), sedentary activity, and experiencing aggression and bullying (Table 6). In the adjusted analysis (Table 7), the feeling of being alone was associated with being physically assaulted four times or more in the last 12 months (PR = 1.56, CI = 1.52–1.59). For students who did not have more than one close friend, there was an association with the use of cigarettes (PR = 1.24, CI = 1.18–1.20) and illicit drugs (PR = 1.26, CI = 1.18–1.35) at least once in the student’s lifetime. Alcohol experimentation was not significant in the adjusted analysis (PR = 0.783).

Sedentarism had an inverse relationship with the possibility of having close friends for students who commonly sit for more than six hours a day (PR = 0.89, CI = 0.84–0.95). That is, students who stay seated longer have more friends. Not having more than one close friend was associated with bullying (PR = 1.37, CI = 1.31–1.43) and being physically assaulted four times or more in the last 12 months (PR = 1.70, CI = 1.58–1.82). Having trouble sleeping due to worries was also associated with using cigarettes (PR = 1.37, CI = 1.34–1.39), alcohol (PR = 1.40, CI = 1.37–1.42), or illicit drugs (PR = 1.38, CI = 1.35–1.42). Remaining seated for six hours or more per day (PR = 1.24, CI = 1.20 – 1.27), not using condoms in the first act of sexual intercourse (PR = 1.12, CI = 1.09–1.16), bullying (PR = 1.44, CI = 1.41–1.47), and being assaulted four times or more in the last 12 months (PR = 1.60, CI = 1.55–1.65) were also associated with not sleeping due to worries (Table 7).

The students who indicated they experienced the symptoms assessed for with the mental health questionnaire had a higher frequency of cigarette, alcohol, and illicit drug use, ranging from three or more days in the last 30 days. Onset of sexual activity prior to age 15 was inversely related to the feeling of being alone and not having more than one close friend (Table 7).

## 4. Discussion

Mental health outcomes can be affected by many factors [23]. Determining the mental state of an individual is a meticulous process that requires preparation, complex evaluation, and the use of appropriate and individual examination instruments [1]. Hence, the PeNSE does not provide enough data to evaluate the mental health of students. However, it does present three indicators that assess the feeling of being alone, lack of close friendships, and trouble sleeping due to worries, and the frequency of these experiences may suggest poor mental health outcomes. Though these three factors are normal human experiences and can occur several times throughout one’s life, if they recur frequently, they should not be disregarded as common occurrences, as they may indicate symptoms of mental health disorders. The number of close friends one has is another indicator. As people are naturally social, not having more than one close friend can be considered as a symptom of isolation and difficulty with socialization [38,39]. The feeling of being alone can also be a symptom presented by socially excluded adolescents, and the two-way relationship between exclusion and mental health has already been established [40]. Sleep quality is also an important factor for mental health [35], and to have trouble sleeping is associated with mental illness [34] and suicidal ideation [21]. Thus, it should be considered that an adolescent who frequently presents with two or more of these symptoms may experience poor mental health outcomes. Mental health symptoms do not necessarily result in the development of severe clinical pathology, but they can badly impact one’s quality of life [20,21].

These three variables of mental health indicators were associated with experimentation (used at least once in a lifetime) of cigarettes, alcohol, and/or illicit drugs. The relationship between substance use and mental health indicators can be reciprocal; that is, the use of drugs can lead to poor mental health outcomes or the opposite [22]. During adolescence, mental health concerns combined with impulsiveness and the need to belong to a group may influence substance use [11].

Our study indicates that the frequency of drug use was similarly related to mental health indicators. The findings show that not having more than one close friend has been related to adolescents’ alcohol consumption and cigarette smoking on ten or more of the last 30 days. Moderate consumption (during one to nine of the last 30 days), however, had a direct association with the number of close friends; that is, adolescents indulging in moderate alcohol consumption had more than one close friend. Therefore, our results agreed with our research objectives, indicating that use of these substances may increase social inclusion for adolescents, explaining why adolescent substance users have more close friends. However, consuming alcohol on ten or more out of 30 days may be related to the development of alcohol dependence. Furthermore, drinking alcohol to the point of intoxication on ten or more days in the last 30 days was related to the three indicators assessed for in our study. A study conducted in 2018 revealed similar results [38], and more frequent alcohol use has been shown to be associated with social isolation [41].

Additionally, our findings showed that using illicit drugs on ten or more out of the last 30 days was correlated with having no more than one friend and experiencing trouble sleeping due to worries. These results are critical, because, in addition to poor mental health outcomes, heavy alcohol use can have harmful medical consequences as well [41,42]. For example, prior research has noted that heavy alcohol use can lead to memory impairment [43].

Poor mental health outcomes can be the consequence or the cause of substance use and vice versa, affecting both the individual and society [10,13]. For adolescents, substance use can alter neurodevelopment, creating aggressive behaviors and possibly leading to engagement in criminal activities. Moreover, their personal lives, including schooling, can be impaired. Mental health disorders can lead to dropping out of school, and even if the individual graduates with a basic education, he/she will generally make less income over a lifetime than those who do not experience poor mental health. Therefore, in addition to the harms the individual may experience as an adolescent, he or she may also experience a lower socioeconomic status as an adult. Usually, the expenses incurred are related to mental health and substance abuse treatment, and while they can be paid for by the individual’s family, they may also impact the state. Furthermore, substance use has been associated with the reduction of labor, increased crime, poverty, unemployment, and early mortality, as well as the development of mental health disorders such as depression and schizophrenia [22]. In addition, substance use has been associated with other physical health concerns such as obesity [44].

Regarding adolescent sexual behaviors, our findings showed that early onset of sexual activity was inversely related to feeling alone and having only one close friend; most likely because, when they are in relationships, adolescents establish bonds of affection. Additionally, the findings of our study were in agreement with other research findings indicating that, although adolescents are aware of the risks from engaging in unprotected sexual intercourse, many still choose not to use condoms [45,46]. Furthermore, prior research has indicated that the use of condoms during the first act of sexual intercourse favors the regularity of the behavior [46]. Some factors may increase the occurrence of nonusage of condoms, such as drug use and mental health disorders [45]. Unprotected sexual intercourse may result in the transmission of sexually transmitted infections and/or adolescent pregnancy, both of which have been shown to increase the likelihood of poor mental health outcomes, such as depression [47].

Not engaging in regular physical activity is also a harmful behavior; mental health and sedentarism have a reciprocal relationship; one shows influence on the other and vice versa. [25]. In our study, we noted that, generally, sitting for six hours or more in a day is associated with feeling alone and experiencing trouble sleeping due to worries. However, our findings also showed that being sedentary was positively associated with having more friends. It is possible that, while sedentary, adolescents engage in activities such as using a computer, playing videogames, and/or social networking, establishing friendships in the process [48].

Our study also found that being a victim of bullying and being physically assaulted was also associated with not having more than one close friend, feeling alone, and having trouble sleeping due to worries. Another study, carried out from 2014 to 2015, analyzed students from Vietnam and identified an association between experiencing bullying and depressive symptoms and suicidal ideation [24]. Aggressive behavior has also been reported as a behavioral change indicative of a mental illness manifestation [49]. Violence and victimization have been shown to be related to poor mental health outcomes as well [50].

Our study has some limitations. First, our findings do not infer causality. Moreover, these datasets are based on self-reports, and the questionnaire was developed specifically for this Brazilian National Survey. The findings may be influenced by social self-perception. It is important to note that PeNSE did not measure psychiatric disorders. We evaluated indicators of mental health from issues related to insomnia and loneliness. Though our survey data was collected in 2015, it is the most recent population survey representative of Brazilian adolescents. Despite the limitations, our analysis showed that the three studied variables (“feeling alone”, “not having more than one close friend”, and “to have trouble sleeping due to worries”), which are indicative of poor mental health outcomes, are related to suggested risky behaviors (substance use, not using a condom during the first act of sexual intercourse, leading a sedentary lifestyle, and being a victim of physical violence and bullying). In this sense, these variables that are related to other public health problems and impact the mental health not only of adolescents but of their family nuclei need to be further investigated. It is also important to understand the role of the school and the family in promoting a healthy environment for mental health. Consequently, it is valid to say that Brazilian students need actions that are focused on discussing poor mental health outcomes and risk behaviors, because these problems are present in the population and are related to each other.

## 5. Conclusions

Drug use causes many problems, mainly related to crime. Being the target of violence and sedentarism can also cause harm to the subject and entail many expenses related to clinical care. Not using condoms during sex can lead to other problems, such as sexually transmitted infections and unplanned pregnancy. In our study, these behaviors were associated with at least one mental illness symptom. Therefore, it is suggested that further studies are developed to understand more aspects of this relationship between risk behaviors and mental health. We also suggest that interdisciplinary initiatives be implemented in schools. An interesting proposal is to talk about mental health in the classroom and present data on risky behaviors and their consequences. After all, an adolescent is less likely to start using drugs or not worry about his mental health if he knows that his psychological and cognitive functions can be adversely affected and, perhaps, permanently affected. So, we hope this study will inspire new studies and intervention actions to help the Brazilian context.

## Figures and Tables

**Table 1:** Questions used to access the indicators of mental health.

Questions	Possible Answer Options
In the last 12 months, how often have you felt alone?	(1) Never or rarely (2) Sometimes (3) Most often or always
In the last 12 months, how often did you not get to sleep at night because something was worrying you a lot?	(1) Never or rarely (2) Sometimes (3) Most often or always
How many close friends do you have?	(1) Up to one friend (2) Two friends or more

**Table 2:** Questions used to access risky behavioral factors.

Risky Behavioral Factors	Specific Assessment Questions
Use of cigarettes	Have you ever smoked cigarettes, even a puff or two? In the last 30 days, on how many days did you smoke cigarettes?
Alcohol consumption	Have you consumed alcoholic beverages during your lifetime? In the last 30 days, on how many days did you drink alcoholic beverages? Did you drink until you became drunk?
Use illicit drugs	Have you ever used illicit drugs in your lifetime? In the last 30 days, on how many days did you use illicit drugs?
Age at onset of sexual activity and condom use	How old were you when you engaged in your first act of sexual intercourse? Did you use a condom during your first act of sexual intercourse?
Being bullied and/or experiencing aggression	Have you ever suffered bullying? How many times were you physically attacked in the last 12 months?
Sedentarism	On a normal day, how many hours do you spend sitting?

**Table 3:** Sample description of the present study and prevalence of a loneliness feeling, number of close friends, and insomnia due to worries among 9th grade adolescents in the 2015 PeNSE (N = 102,072).

Variables	Total		Feel Alone			Does Not Have More Than 1 Close Friend			Have Trouble Sleeping Due to Worries		
	N	%	N	%	*p*	N	%	*p*	N	%	*p*
**Gender**					<0.001			0.016			<0.001
Male	49,290	48.3	16,035	32.7		5026	10.3		13,062	26.7	
Female	52,782	51.7	29,427	55.9		5620	10.7		23,731	45.1	
**Age**											
≤13 y.o.	17,260	16.9	7487	43.5	<0.001	1488	8.6	<0.001	5666	32.9	<0.001
14 y.o.	51,611	50.6	22,839	44.4		5010	9.7		18,070	35.1	
15 y.o.	20,864	20.4	9382	45.2		2452	11.8		7978	38.5	
16 y.o. or more	12,337	12.1	5754	47.0		1696	13.9		5079	41.6	
**Mother’s Education**										
Did not study	5531	5.4	2622	47.6	<0.001	802	14.6	<0.001	2297	41.8	<0.001
Started primary education	24,241	23.7	11,075	45.8		2748	11.4		9009	37.3	
Started secondary school	24,178	23.7	10,811	44.8		2421	10.0		8577	35.6	
Started post-secondary school	22,688	22.2	10,036	44.3		1897	8.4		7902	34.9	

PeNSE: Pesquisa Nacional de Saúde do Escolar (National School Health Survey).

**Table 4:** Results of bivariate analysis between substance use and mental health indicators among 9th grade adolescents in the 2015 PeNSE.

Variables	Feel Alone			Does Not Have More than 1 Close Friend			Have Trouble Sleeping Due to Worries		
	%	PR	*p*	%	PR	*p*	%	PR	*p*
**Smoked cigarettes**									
No	42.4	1	<0.001	9.9	1	<0.001	33.9	1	<0.001
Yes	54.9	1.30 (1.28–1.32)		12.9	1.31 (1.25–1.36)		46.6	1.38 (1.35–1.40)	
**In the last 30 days, how many days did you smoke cigarettes**									
None	54.2	1	<0.001	12.7	1	<0.001	44.4	1	<0.001
1 to 2 days	56.6	1.04 (1.01–1.02)		12.4	0.98 (0.88–1.09)		51.0	1.15 (1.10–1.20)	
3 to 9 days	59.8	1.10 (1.05–1.16)		12.2	0.96 (0.83–1.12)		54.1	1.22 (1.16–1.29)	
10 or more	54.8	1.01(0.96–1.07)		17.8	1.41 (1.24–1.60)		52.8	1.19 (1.13–1.26)	
**Have you been consumed alcoholic beverages during your lifetime**									
No	38.2	1	<0.001	10.3	1	0.049	29.5	1	<0.001
Yes	50.8	1.33 (1.31–1.35)		10.7	1.04 (1.00–1.06)		42.4	1.44 (1.41–1.46)	
**In the last 30 days, how many days did you drink alcoholic beverages**									
None	48.5	1	<0.001	10.9	1	<0.001	38.4	1	<0.001
1 to 2 days	52.8	1.09 (1.07–1.11)		10.2	0.94 (0.88–0.99)		45.6	1.19 (1.16–1.22)	
3 to 9 days	54.0	1.11(1.08–1.14)		10.0	0.91 (0.84–0.99)		48.9	1.27 (1.24–1.31)	
10 or more	53.5	1.10 (1.06–1.14)		12.6	1.16 (1.05–1.29)		53.9	1.40 (1.35–1.46)	
**Did you drink until you became drunk**									
None	47.9	1	<0.001	10.2	1	<0.001	38.4	1	<0.001
1 to 2 days	55.0	1.15 (1.13–1.17)		11.2	1.09 (1.03–1.16)		46.9	1.22 (1.20–1.25)	
3 to 9 days	55.0	1.15 (1.12–1.18)		12.0	1.18 (1.08–1.28)		51.3	1.34 (1.30–1.38)	
10 or more	52.8	1.10 (1.06–1.15)		12.5	1.23 (1.09–1.37)		51.6	1.34 (1.29–1.40)	
**Used illicit drugs in your lifetime**									
No	43.8	1	<0.001	10.2	1	<0.001	35.0	1	<0.001
Yes	54.8	1.26 (1.23–1.28)		13.5	1.32 (1.25–1.40)		49.0	1.40 (1.37–1.43)	
**In the last 30 days, how many days did you use illicit drugs**									
None	53.7	1	0.023	13.6	1	0.002	47.0	1	<0.001
1 to 2 days	57.2	1.07 (1.02–1.12)		12.2	0.90 (0.78–1.03)		49.8	1.06 (1.01–1.12)	
3 to 9 days	56.6	1.05 (0.99–1.12)		12.5	0.92 (0.78–1.10)		54.4	1.16 (1.09–1.23)	
10 or more	52.9	0.98 (0.92–1.06)		17.4	1.28 (1.08–1.51)		51.3	1.09 (1.02–1.18)	

PeNSE: Pesquisa Nacional de Saúde do Escolar (National School Health Survey), PR: prevalence ratio, and CI: confidence interval.

**Table 5:** Results of bivariate analysis between sexual behavior and mental health indicators of bivariate analysis among 9th grade adolescents in the 2015 PeNSE.

Variables	Feel Alone			Does Not Have More Than 1 Close Friend			Have Trouble Sleeping Due to Worries		
	%	PR	*p*	%	PR	*p*	%	PR	*p*
**How old were you during your first act of sexual intercourse**									
15 y.o. or more	48.9	1	<0.001	13.8	1	<0.001	44.5	1	<0.001
13 and 14 y.o.	46.1	0.94 (0.91–0.97)		11.3	0.82 (0.76–0.89)		41.8	0.94 (0.91–0.97)	
12 y.o. or less	42.7	0.87 (0.84–0.91)		13.0	0.94 (0.86–1.03)		36.1	0.81 (0.79–0.85)	
**Use of condom during first act of sexual intercourse**									
Yes	43.9	1	<0.001	11.3	1	<0.001	39.6	1	<0.001
No	50.9	1.16 (1.13–1.19)		14.2	1.26 (1.18–1.34)		44.6	1.13 (1.09–1.16)	

PeNSE: Pesquisa Nacional de Saúde do Escolar (National School Health Survey), PR: prevalence ratio;, and CI: confidence interval.

**Table 6:** Results of bivariate analysis risk behaviors and mental health indicators of bivariate analysis among 9th grade adolescents in the 2015 PeNSE.

Variables	Feel Alone			Does Not Have More Than 1 Close Friend			Have Trouble Sleeping Due to Worries		
	%	PR	*p*	%	PR	*p*	%	PR	*p*
**In an average day, how many hours do you spend sitting**									
Up to 1 h	40.2	1	<0.001	13.0	1	<0.001	34.7	1	<0.001
1 to 3 h	40.8	1.02 (0.99–1.04)		9.9	0.76 (0.73–0.81)		32.7	0.94 (0.92–0.97)	
3 to 6 h	45.1	1.12 (1.10–1.15)		9.3	0.72 (0.68–0.75)		35.4	1.02 (1.00–1.05)	
>6 h	52.3	1.30 (1.28–1.33)		10.4	0.80 (0.76–0.85)		42.3	1.22 (1.19–1.25)	
**Have you ever suffered bullying**						<0.001			
No	34.8	1	<0.001	8.9	1		29.5	1	<0.001
Yes	55.3	1.59 (1.57–1.61)		12.0	1.34 (1.29–1.39)		43.2	1.47 (1.44–1.49)	
**How many times were you physically attacked in the last 12 months**									
None	41.3	1	<0.001	9.9	1	<0.001	33.3	1	<0.001
Once	58.2	1.41 (1.38–1.42)		11.7	1.19 (1.11–1.26)		47.2	1.42 (1.38–1.45)	
2 to 3 times	62.2	1.51 (1.47–1.54)		12.3	1.24 (1.15–1.35)		50.6	1.52 (1.47–1.56)	
4 or more times	62.0	1.50 (1.47–1.53)		15.7	1.59 (1.49–1.69)		51.8	1.55 (1.51–1.60)	

PeNSE: Pesquisa Nacional de Saúde do Escolar (National School Health Survey), PR: prevalence ratio, and CI: confidence interval.

**Table 7:** Multivariate analysis adjusted by gender, age, and mother’s education.

Variables	Feel Alone		Does Not Have More Than 1 Close Friend		Have Trouble Sleeping Due to Worries	
	PR Adjusted	*p*	PR Adjusted	*p*	PR Adjusted	*p*
**Have you smoked cigarettes during your lifetime**						
No	1	<0.001	1	<0.001	1	<0.001
Yes	1.31 (1.29–1.33)		1.24 (1.18–1.20)		1.37 (1.34–1.39)	
**In the last 30 days, how many cigarettes did you smoke**						
None	1	<0.001	1	<0.001	1	<0.001
1 to 2 days	1.02 (0.98–1.06)		0.94 (0.83–1.07)		1.08 (1.03–1.13)	
3 to 9 days	1.11 (1.06–1.17)		0.94 (0.78–1.12)		1.22 (1.15–1.30)	
10 or more	1.05 (0.99–1.12)		1.47 (1.27–1.70)		1.24 (1.17–1.33)	
**Have you been consumed alcohol during your lifetime**						
No	1	<0.001	1	0.783	1	<0.001
Yes	1.31 (1.29–1.33)		1.01 (0.6–1.05)		1.40 (1.37–1.42)	
**In the last 30 days, how many days did you drink alcoholic beverages**						
None	1	<0.001	1	<0.001	1	<0.001
1 to 2 days	1.06 (1.04–1.08)		0.91 (0.85–0.98)		1.14 (1.11–1.17)	
3 to 9 days	1.11 (1.08–1.14)		0.90 (0.82–0.99)		1.27 (1.23–1.31)	
10 or more	1.13 (1.08–1.17)		1.16 (1.04–1.31)		1.41 (1.35–1.47)	
**Did you drink until drunk**						
None	1	<0.001	1	<0.001	1	<0.001
1 to 2 days	1.14 (1.11–1.16)		1.06 (0.99–1.14)		1.20 (1.18–1.24)	
3 to 9 days	1.17 (1.13–1.21)		1.16 (1.06–1.28)		1.36 (1.31–1.40)	
10 or more	1.17 (1.12–1.23)		1.17 (1.02–1.34)		1.42 (1.35–1.49)	
**Have you been used illicit drugs during your lifetime**						
No	1	<0.001	1	<0.001	1	<0.001
Yes	1.26 (1.23–1.29)		1.26 (1.18–1.35)		1.38 (1.35–1.42)	
**In the last 30 days, how many days did you use illicit drugs**						
None	1	0.195	1	0.003	1	<0.001
1 to 2 days	1.05 (1.00–1.10)		0.89 (0.76–1.04)		1.05 (0.99–1.11)	
3 to 9 days	1.05 (0.99–1.12)		0.97 (0.80–1.17)		1.16 (1.09–1.25)	
10 or more	1.04 (0.96–1.13)		1.34 (1.11–1.62)		1.20 (1.11–1.30)	
**In a normal day, how many hours do you spend sitting**						
Up to 1 h	1	<0.001	1	<0.001	1	<0.001
1 to 3 h	1.02 (0.99–1.05)		0.81 (0.76–0.86)		0.97 (0.94–1.00)	
3 to 6 h	1.13 (1.11–1.16		0.79 (0.75–0.84)		1.07 (1.04–1.10)	
6 h or more	1.29 (1.26–1.32)		0.89 (0.84–0.95)		1.24 (1.20–1.27)	
**How old were you in your first act of sexual intercourse**						
15 years or more	1	<0.001	1	<0.001	1	0.509
13 and 14 years	0.92 (0.89–0.96)		0.79 (0.72–0.86)		0.98 (0.94–1.02)	
12 years or less	0.91 (0.87–0.96)		0.85 (0.76–0.96)		0.97 (0.92–1.03)	
**Use of condom in first act of sexual intercourse**						
Yes	1	<0.001	1	<0.001	1	<0.001
No	1.15 (1.11–1.18)		1.26 (1.17–1.36)		1.12 (1.09–1.16)	
**Suffered bullying**						
No	1	<0.001	1	<0.001	1	<0.001
Yes	1.56 (1.54–1.59)		1.37 (1.31–1.43)		1.44 (1.41–1.47)	
**How many times were you physically attacked in the last 12 months**						
None	1	<0.001	1	<0.001	1	<0.001
Once	1.41 (1.38–1.44)		1.25 (1.16–1.34)		1.40 (1.36–1.44)	
2 to 3 times	1.50 (1.46–1.54)		1.29 (1.17–1.41)		1.54 (1.49–1.59)	
4 or more times	1.56 (1.52–1.59)		1.70 (1.58–1.82)		1.60 (1.55–1.65)	

PeNSE: Pesquisa Nacional de Saúde do Escolar (National School Health Survey), PR: prevalence ratio, and CI: confidence interval
